# Psychopathological Symptoms and Well-Being in Overweight and Underweight Adolescents: A Network Analysis

**DOI:** 10.3390/nu13114096

**Published:** 2021-11-16

**Authors:** Michael Zeiler, Julia Philipp, Stefanie Truttmann, Karin Waldherr, Gudrun Wagner, Andreas Karwautz

**Affiliations:** 1 Eating Disorder Unit, Department for Child and Adolescent Psychiatry, Medical University of Vienna, 1090 Vienna, Austria; julia.philipp@meduniwien.ac.at (J.P.); stefanie.truttmann@meduniwien.ac.at (S.T.); gudrun.wagner@meduniwien.ac.at (G.W.); andreas.karwautz@meduniwien.ac.at (A.K.); 2 Department for Research and Development, Ferdinand Porsche FernFH-Distance Learning University of Applied Sciences, 2700 Wiener Neustadt, Austria; karin.waldherr@fernfh.ac.at

**Keywords:** overweight, obesity, underweight, adolescents, mental health, psychopathology, quality of life, eating disorder risk, network analysis

## Abstract

Overweight and underweight adolescents have an increased risk of psychological problems and reduced quality of life. We used a network analysis approach on a variety of psychopathology and well-being variables to identify central factors in these populations. The network analysis was conducted on data of 344 overweight adolescents (>90th BMI-percentile) and 423 underweight adolescents (<10th BMI-percentile) drawn from a large community sample (10–19 years) including behavioral and emotional problems (Youth Self-Report), eating disorder risk (SCOFF) and well-being variables (KIDSCREEN). Additionally, psychopathology and well-being scores of overweight and underweight individuals were compared with 1.560 normal weight adolescents. Compared to their normal weight peers, overweight adolescents showed elevated psychopathology and eating disorder risk as well as reduced well-being. Underweight adolescents reported increased levels of internalizing problems but no increased eating disorder risk or reduced well-being. The network analysis revealed that anxious/depressed mood and attention problems were the most central and interconnected nodes for both overweight and underweight subsamples. Among underweight individuals, social problems and socially withdrawn behavior additionally functioned as a bridge between other nodes in the network. The results support psychological interventions focusing on improving mood, coping with negative emotions and tackling inner tension.

## 1. Introduction

It is well known that overweight or obese children and adolescents are at increased risk of psychopathological symptoms, behavioral and emotional problems as well as reduced quality of life. Previous evidence shows elevated symptoms of depression [1,2,3], anxiety [2] and conduct disorders [4], more emotional difficulties and peer problems [3], lower self-esteem [5] as well as higher school absenteeism [6] for overweight or obese children and adolescents compared to their normal weight peers. A higher prevalence of disordered eating, particularly binge eating behavior, and reduced body satisfaction were found in overweight/obese adolescents [7,8,9]. Furthermore, reduced quality of life in physical, mental and social domains was consistently reported [10,11].

While less intensively discussed in the literature, children and adolescents at the lower end of the weight spectrum have also become a focus of attention. Apart from disordered eating [12] and body dissatisfaction [13] reported in this subgroup (which may represent problems indicating symptoms of anorexia nervosa), internalizing problems in particular, including depression and socially withdrawn behavior, were reported for underweight adolescents [14,15]. Moreover, previous studies have shown that weight-related teasing occurs for overweight and underweight adolescents, which consequently may increase the risk of social isolation and mental health problems for both groups [16,17].

Due to these multiple mental health concerns, psychological factors have been identified as an important target for selective prevention and treatment of obesity in adolescents [18] but also in underweight adolescents. Some authors even point to integral prevention and intervention approaches for individuals at both ends of the weight spectrum because of shared environmental risk factors for (severe) underweight and obesity, which, for example, include teasing, peer problems and negative family relationships [19,20,21]. Of note, in the latest Cochrane review on dietary and physical activity interventions for preventing obesity in youths, including more than 150 randomized-controlled trials, only a few interventions included components targeting psychological factors such as depression, anxiety, self-esteem, support by peers, stress and body image [22]. This is all the more interesting because another review focusing on psychological interventions for overweight or obese individuals revealed significant improvements in depression, self-esteem, body-image, anxiety, stress, disordered eating and general well-being while reaching similar weight loss compared to dietary and physical activity interventions directly targeting the weight [23].

Thus, there is evidence that psychological interventions for adolescents with weight-related problems should be provided. However, due to the variety of psychological problems and well-being variables associated with overweight and underweight, which also opens up a variety of possible targets for prevention and treatment interventions, one may ask whether there exist specific ‘core’ mental health domains that such interventions should focus on and that may be most beneficial regarding the intervention outcomes. One method to tackle this question is psychological network analysis. A network analysis is a relatively new statistical approach to model the complex interactions between a large number of different variables (called ‘network’). It allows the identification of specific associations between variables in the network on the one hand, and the identification of central variables on the other hand (c.f. [24]). In brief, a central variable in a network is highly associated with other variables in the network. Thus, it can be assumed that if a central variable is changed, this also has effects on many other variables in the network whereby these effects are usually not directly proportional. Consequently, a network analysis on psychopathological symptoms and well-being variables obtained in overweight and underweight adolescents should help identifying central symptoms and characteristics. For example, if body dissatisfaction turns out to be a central variable in a network including different mental health problems in overweight adolescents, this variable would be a promising target for interventions as reducing body dissatisfaction would presumably also affect other psychopathological symptoms in the network (e.g., depression and anxiety).

In recent years, psychological network analyses have been performed in patients with diagnosed eating disorders including anorexia nervosa and bulimia nervosa with the aim to identify central symptoms of the eating disorder pathology. Studies including a variety of eating disorder symptoms in their network analysis found that shape and weight concerns, desiring weight loss, desire to be thinner, feeling ineffective, worries that feeling will get out of control and guilt after overeating were the most central symptoms [25,26,27]. Other studies on eating disorder patients which additionally included general psychopathology showed that depressive and anxiety symptoms, interpersonal sensitivity and personal alienation had the highest centrality in the network [28,29]. Authors of these studies concluded that these symptoms represent important targets for effective treatment. Studies using a network approach on mental health in overweight and obese individuals are scarce and have focused on adult individuals only. Calugi and Dalle Grave [30] reported that interpersonal sensitivity and shape-weight concerns were the most central variables in adult patients with obesity, while disordered eating symptoms including binge eating and dietary restraint were the most peripheral and least connected symptoms in the network. Another network analysis on physical performance and quality of life variables emphasized the importance of mental health as a key factor in adults with obesity [31]. Moreover, in a very small sample of obese children, aspects of unhealthy eating behavior, physical activity habits and low mood turned out to be central variables [32]. In another network analysis among a general sample of adolescents including different variables on executive function and disinhibited eating, emotional eating emerged as the most central symptom [33]. So far, no study has used a network analysis approach to explore the interconnection of psychopathology and quality of life variables obtained in overweight/obese or underweight adolescents.

Thus, the present study has the following aims: First, we aimed to investigate psychopathological symptoms and well-being/quality of life in overweight or obese adolescents from a large representative community sample. We hypothesized that overweight or underweight adolescents would show higher levels of psychopathology and reduced well-being compared to their normal weight peers. Second, using a network analysis approach we aimed to identify central factors among a variety of psychopathological symptoms and well-being variables, which will inform about potential beneficial targets for psychological interventions (e.g., indicated prevention approaches) for overweight and underweight adolescent populations.

## 2. Materials and Methods

### 2.1. Sampling and Recruitment

In this study, we used data from the ‘Mental Health in Austrian Teenagers’ (MHAT, [34]) study, an epidemiological survey that aimed to obtain the prevalence of mental health problems in a large representative sample of Austrian adolescents aged 10 to 18 years. The main part of the sample was recruited via schools (*n* = 261 schools, including all school types in all regions of Austria). School classes of the 5th, 7th, 9th and 11th grade were randomly selected from participating schools and all students within these classes were invited to participate. A total of 3.615 adolescents from the school sample participated in this study (response rate: 47.3%). The participants completed a comprehensive questionnaire to obtain sociodemographic information, behavioral and emotional problems and well-being/quality of life (see Section 2.2). The school sample was complemented by a small sample of adolescents who dropped out of school and who were recruited from training courses for unemployed adolescents (*n* = 43, 1.1% of the total sample) and by a small sample of adolescents currently in inpatient treatment due to a psychiatric disorder who were recruited from child and adolescent psychiatry wards across Austria (*n* = 133, 3.5% of the total sample). This was done to also cover adolescents from the population who cannot be reached via the regular school setting (due to early school dropout and severe mental health problems). This sample composition reflects the general population of Austrian adolescents including all levels of psychopathological symptoms and quality of life. Thus, this sample allows to adequately tackle the main research question of this paper (identifying central mental health aspects that may be promising targets for indicated prevention strategies for overweight and underweight adolescent populations). Written informed consent was collected from all participants and legal representatives prior to the inclusion in the study. Ethical approval was obtained from the Ethics Committee of the Medical University of Vienna (#1134/2013). Details about the sampling, recruitment strategy and procedures are published in Zeiler et al. [34] and Wagner et al. [35].

For the purpose of this study, we used subsamples of the entire dataset and included overweight adolescents defined by body-mass index (BMI) ≥ 90th sex and age specific percentile (*n* = 344) as well as underweight adolescents defined by BMI ≤ 10th sex and age specific percentile (*n* = 423). Data from normal weight adolescents (25th < BMI percentile > 75th, *n* = 1.560) were used as a reference to enable a classification of psychopathology and well-being scores of the overweight/underweight subsamples. Weight and height measures were derived from the adolescents’ self-reports. Participants who did not provide any (valid) height/weight information were excluded.

### 2.2. Instruments

Apart from sociodemographic information (e.g., sex, age, migration background, living situation, diagnosed somatic and psychiatric disorders in the family) that was used to describe the sample, we obtained data from three validated and often used instruments to assess psychopathological symptoms and quality of life. The strength of a network analysis lies in exploring complex associations among a large number of diverse psychological features. Thus, we selected instruments that assess many different aspects of psychopathology and quality of life in a dimensional/continuous way. While network estimation approaches to handle categorical and ordinal data exists, such data types are still regarded as suboptimal [36]. Moreover, the selection of instruments was driven by the limited time provided by the schools to complete the entire questionnaire battery (max. one school hour of 50 min).

Specifically, data obtained through the following instruments were included in the network analysis:

The Youth Self-Report (YSR, [37,38]) is a widely used self-report instrument to measure a wide range of behavioral and emotional problems (112 items rated on a three-point scale). Item ratings are summed up in eight syndrome scales (‘socially withdrawn’, ‘somatic complaints’, ‘anxious/depressed’, ‘social problems’, ‘thought problems’, ‘attention problems’, ‘dissocial behavior’ and ‘aggressive behavior’). Additionally, the items can be aggregated to three broadband scales, a total problem score, an internalizing problem score and an externalizing problem score. The YSR raw scores were used in this study. However, we also calculated the percentage of clinically relevant problem scores by using the available norms (cut-off: *T*-score > 63 for broadband scales). Internal consistencies were excellent for the broadband scales (Cronbach’s alpha > 0.86) and acceptable for the syndrome scales (Cronbach’s alpha between 0.56–0.86).

We used the SCOFF questionnaire [8,39] to screen for eating disorders, an aspect that is not covered in the YSR questionnaire but which is particularly relevant for overweight and underweight populations). It assesses five core features of eating disorders, including significant weight loss, intentional vomiting, body dissatisfaction, loss of control over food and food intrusive thought. The presence/absence of each symptom is rated on a dichotomous scale (‘yes’ vs. ‘no’). The total score representing the number of present eating disorder symptoms (possible score range 0–5) was used in the present study. According to the authors of the original SCOFF version [39], a score ≥ 2 represents an increased risk for eating disorders. A recently published meta-analysis reported a pooled sensitivity of 86% and specificity of 83% using full-syndrome eating disorders or other established eating disorder questionnaires as reference [40].

Moreover, we used the KIDSCREEN scales [41] to obtain well-being and quality of life in different domains including ‘self-perception’ (satisfaction with own body and appearance), ‘parent relation and home life’ (assessing the quality of relationship with parents, feeling understood by them, being able to talk with them), ‘social support and peers’ (assessing the quality of peer relationship, spending joyful time with friends, helping each other, being able to rely on friends), ‘school environment’ (satisfaction with the school environment, getting along well with teachers, being able to concentrate well) and ‘social acceptance’ (assessing the absence of bullying). Items are rated on a five-point scale; higher subscale scores indicate higher levels of well-being/quality of life. In addition to the subscales, a general measure of well-being (‘KIDSCREEN-10′) was calculated. In the present study, the raw scores of the general and subscale measures were used. Internal consistencies of the scales ranged from 0.77 to 0.89.

### 2.3. Data Analysis

Descriptive analyses and comparative analyses of the overweight, underweight and normal weight subsamples were performed using IBM SPSS Statistics 27.0. The network analysis was conducted using JASP (version 0.12.2.0) [42] which makes use of the *R* packages ‘*bootnet*’ [43] and ‘*qgraph*’ [44].

First, we compared YSR, SCOFF and KIDSCREEN (general and subscale) scores obtained from the overweight and underweight subsamples with the normal weight reference group using general linear models controlling for sex. Differences between the overweight/underweight and normal weight samples were analyzed using Tukey tests. We further used Chi²-tests to compare the percentages of clinically relevant YSR scores and eating disorder risk (SCOFF ≥ 2) between the overweight, underweight and normal weight reference samples.

The network analysis for overweight and underweight adolescents was performed on general psychopathology variables (YSR syndrome scales), eating disorder risk (SCOFF total score) and well-being/quality of life variables (KIDSCREEN scores, not using the KIDSCREEN-10 general quality of life score). Due to the correlational nature of this approach and as age-/sex-standardized scores are not available for all of the included instruments, we used the raw scores of these questionnaires in this analysis. A network is defined as a set of variables (called ‘nodes’) which are reciprocally connected through ‘edges’ (most commonly some kind of correlation) that do not imply a priori direction or allow causal inference. In the present study, we estimated partial correlation networks using the graphical Least Absolute Shrinkage and Selection Operator (gLASSO [45]). Using the gLASSO estimation, small or unstable correlations within the network are set to zero, resulting in a more parsimonious and better interpretable network only depicting the most robust associations between the nodes. Each edge represents the thus regularized partial correlation between two nodes. In contrast to non-regularized partial correlations (all edges between all nodes are estimated and included in the network plot), regularized partial correlations are used to effectively assess the sparse and interpretable network structure. The stronger the partial correlation between two nodes (either positive or negative), the thicker the edge presented in the network plot. As gLASSO produces a collection of network solution, the Extended Bayesian Information Criterion (EBIC, [46]) was used to select the optimal network model. The Fruchterman–Reingold algorithm [47] was used to organize the network plot. Nodes with more or stronger connections are placed closer together while nodes with less connection are placed further apart.

The centrality of the nodes was estimated with the node strength, betweenness and closeness centrality indices. Node ‘strength’ refers to the weighted number and strength of all connections of a specific node and thus represents the overall influence of a node in the network. ‘Betweenness’ represents the number of shortest paths that pass through the node of interest, respectively, the number of times that the node represents the shortest path between other nodes; thus, a node with high betweenness is important in the connection that other nodes have between them (node acting as a bridge). ‘Closeness’ quantifies the number of direct and indirect links between the node of interest to all other nodes in the network; thus, a node with high closeness will be affected quickly by changes in any part of the network and vice versa (c.f. [24]). *z*-Standardized centrality indices (mean = 0, SD = 1) are reported.

Moreover, we performed the Network Comparison Test (NCT, [48]) using the ‘*NetworkComparisonTest*’ package in *R* to compare the network structure and the global network strength between the overweight and underweight samples.

### 2.4. Sample Size Considerations and Network Stability/Edge Accuracy Calculations

Currently, there is no established method for a formal power analysis available for psychological network analyses, nor is there a minimal sample size required for this type of analysis [43]. Rather, there are established methods to evaluate the network stability (e.g., stability of central indices) and edge accuracy which should be reported along a psychological network analysis. Providing evidence for the stability of a network solution is an important prerequisite to reasonably interpret the network. In general, there is a larger chance to find stable network solutions in larger samples than in smaller samples.

The accuracy of the network solution was evaluated by the two following analyses: First, the case-dropping subset bootstrap approach was used to analyze the stability of central indices after observing only subsamples of the data. The correlation stability (CS) coefficient quantifies the stability of central indices and represents the maximum proportion of cases that can be dropped from the full dataset so that the correlation between the original central indices and central indices calculated from bootstrap subsets has a 95% probability of being *r* = 0.7 or higher. Ideally, the CS-coefficients should be above 0.5 [43]. Second, the accuracy of edge weights was evaluated by calculating 95% confidence intervals based on non-parametric bootstrapping (*n* = 1.000 boots) which is recommended for LASSO regularized edges [43]. In case of excessively large bootstrapped confidence intervals, the edge strengths should be interpreted with caution.

## 3. Results

### 3.1. Sample Description

Key characteristics of the overweight and underweight subsamples in reference to adolescents with normal weight are provided in Table 1. Compared to the overweight and normal weight samples, there were more females in the underweight group. The percentage of adolescents with migration background was highest among overweight adolescents. Moreover, the percentage of adolescents with parents where both are employed was lowest in the overweight subsample. Compared to the normal weight reference group, the percentage of adolescents with any diagnosed psychiatric disorder was elevated in the overweight and underweight subsamples. The mean BMI of the overweight group was 27.00 (1.83 standard deviations above the BMI expected according to sex and age). The mean BMI of the underweight group was 15.67 (1.92 standard deviations below the BMI expected according to sex and age).

### 3.2. Psychopathology and Well-Being of Overweight and Underweight Adolescents

Compared to normal weight adolescents, overweight adolescents showed significantly higher YSR total and internalizing scores as well as higher levels of psychopathology in most YSR subscales (except in those related to the externalizing problem domain) (Table 2). Moreover, overweight adolescents reported significantly more symptoms of eating disorders and lower levels of well-being in all domains compared to the normal weight group. Underweight adolescents showed significantly higher scores in the YSR internalizing problem domain and the socially withdrawn subscale compared to the normal weight group. Regarding externalizing problems (including the dissocial and aggressive behavior subscales) and the SCOFF, underweight adolescents even reported problem scores significantly lower than normal weight adolescents. There were no other statistically significant differences between the underweight and normal weight group.

Considering the established cut-off scores for the YSR instrument, 26.7% of overweight adolescents showed clinically relevant total problem scores which was a significantly higher percentage than in the underweight (15.6%) and normal weight (16.2%) subsamples (Chi²(2) = 22.927, *p* < 0.001). Clinically relevant internalizing problems were reported in 24.7% of overweight adolescents which was similar to underweight adolescents (22.3%) but higher than in the normal weight reference group (19.0%) (Chi²(2) = 6.841, *p* < 0.033). A significantly lower number of underweight adolescents (5.5%) showed clinically relevant externalizing problems compared to overweight (10.5%) and normal weight adolescents (8.8%) (Chi²(2) = 6.953, *p* = 0.031). Eating disorder risk (defined as SCOFF score ≥ 2) was reported in 41.3% of overweight adolescents which was significantly higher than in the underweight (16.5%) and normal weight (23.1%) sample (Chi²(2) = 68.189, *p* < 0.001).

### 3.3. Results of the Network Analysis

#### 3.3.1. General Network Structure

**Figure 1:**
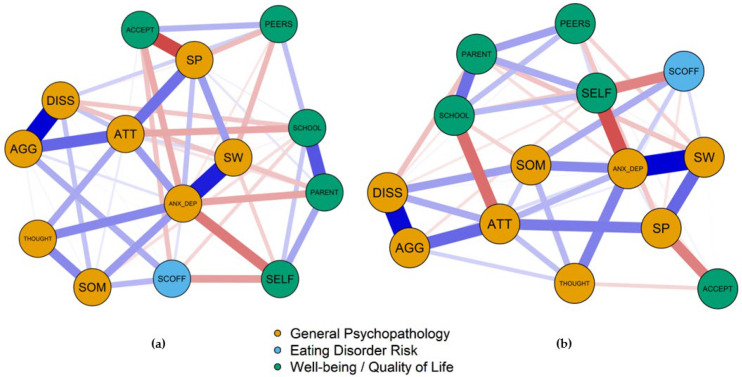
Network plots of the estimated EBIC gLASSO networks of (**a**) overweight adolescents; (**b**) underweight adolescents. Each node represents a variable of the YSR, SCOFF or KIDSCREEN scales. Each link (‘edge’) represents the partial correlation (blue = positive correlation, red = negative correlation). Thicker edges represent stronger associations. Variable abbreviations: ACCEPT Social Acceptance, AGG Aggressive Behavior, ANX_DEP Anxious Depressed, ATT Attention Problems, DISS Dissocial Behavior, PARENT Parent Relation and Home Life, PEERS Social Support and Peers, SCHOOL School Environment, SCOFF SCOFF Score, SELF Self Perception, SOM Somatic Complaints, SP Social problems, SW Socially Withdrawn, THOUGHT Thought Problems.

Figure 1 shows the network plots based on the EBIC gLASSO estimation for the (**a**) overweight and (**b**) underweight group. In the overweight group, anxious/depressed mood is placed very central in the network with strong associations to socially withdrawn behavior and moderate associations to other psychopathological symptoms (e.g., thought problems) and well-being variables (particularly self-perception). Attention problems was another node with several associations to other nodes in the network, especially aggressive behavior and social problems. Aggressive and dissocial behavior were clustered at the periphery of the network. Interestingly, eating disorder risk obtained with the SCOFF was one of the most peripheral nodes in the network. However, it was placed next to self-perception (assessing satisfaction with body and appearance) and somatic complaints. Moreover, nodes related to the contact, problems and satisfaction with peers were plotted next to each other.

In the underweight group, again, anxious/depressed mood was strongly associated to several other nodes in the network including socially withdrawn behavior, self-perception, somatic complaints and thought problems. Attention problems were strongly positively associated with aggressive behavior and social problems and negatively linked to satisfaction with the school environment. With the exception of the social acceptance domain of the KIDSCREEN questionnaire, well-being variables seem to form a cluster within the network, with well-being regarding school being strongly negatively associated with attention problems and self-perception being negatively associated with anxious/depressed mood and eating disorder risk. As in the overweight group, the SCOFF was one of the most peripheral nodes in the network.

#### 3.3.2. Centrality Indices

**Figure 2:**
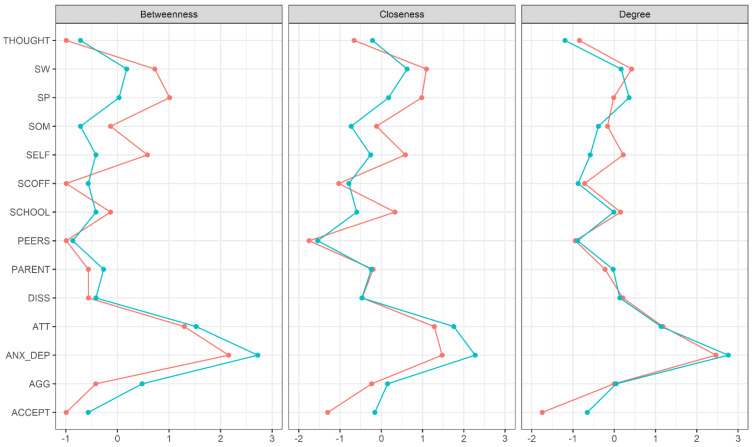
Centrality plot depicting standardized centrality indices (betweenness, closeness, degree = strength) of psychopathological symptoms, eating disorder risk and well-being measures in overweight (blue lines) and underweight (red lines) adolescents. See footnote of Figure 1 for variable abbreviations.

The central indices for all nodes in the network are shown in Figure 2. Anxious/depressed mood (overweight group: *z* = 2.76, underweight group: *z* = 2.47) and attention problems (overweight group: *z* = 1.14, underweight group: *z* = 1.18) were by far the nodes with the highest strength (named ‘degree’ in the centrality plot), thus representing the nodes with the highest overall influence in the network. These nodes also had the highest betweenness (anxious/depresses: *z* = 2.72/*z* = 2.16, attention problems: *z* = 1.53/*z* = 1.30) and closeness (anxious/depresses: *z* = 2.28/*z* = 1.48, attention problems: z = 1.76/*z* = 1.29), indicating that they also function as a bridge between other nodes of the network. For the underweight group, betweenness and closeness was also relatively high for the social problems (betweenness: *z* = 1.01, closeness: *z* = 0.98) and socially withdrawn (betweenness: *z* = 0.73, closeness: *z* = 1.09) nodes. The exact standardized centrality coefficients for all nodes (separated by the overweight and underweight groups) are provided in Supplementary Table S1.

#### 3.3.3. Comparison between Overweight and Underweight Adolescents

The network comparison test revealed no statistically significant difference between the networks for overweight and underweight adolescents regarding the structural invariance (M = 0.118, *p* = 0.967) and the global network strength (S = 0.470, *p* = 0.159).

#### 3.3.4. Stability of Central Indices and Edge Accuracy

For the overweight group, the CS coefficients were 0.75 for strength, 0.67 for betweenness and 0.67 for closeness. For the underweight group, the CS coefficients were 0.75, 0.59 and 0.67, respectively. Furthermore, the correlation stability plots (Figures S1 and S2 in the Supplementary Material) show that the correlation with the original centrality indices decreases slowly when an increasing number of participants are dropped from the full dataset. This indicates that the stability of all centrality indices in both samples are sufficiently high and can be reliably interpreted.

Figures S3 and S4 (Supplementary Material) show the bootstrapped 95% confidence intervals of edge weights for the network of the overweight and underweight groups. The edge accuracy plots reveal that the confidence intervals of edge weights are not excessively large; thus, the estimations of the edge weights seem to be sufficiently accurate to be reasonably interpreted.

#### 3.3.5. Network Structure in Normal Weight Adolescents

While this was not the focus of this study, we additionally estimated the EBIC *g*LASSO network for the normal weight reference group (25th < BMI percentile > 75th) to explore whether the centrality indices are similar to those estimated from the overweight and underweight subsamples and whether the network of psychopathology and well-being variables holds regardless of the adolescent weight status. Indeed, anxious/depressed mood showed the highest strength followed by attention problems and social problems. Regarding betweenness and closeness, attention problems turned out to be by far the most central variable followed by anxious/depressed mood, social problems and satisfaction with the school environment. The network and centrality plots for the normal weight sample are provided in Supplementary Figure S5. Thus, in normal weight adolescents, attention problems as well as satisfaction with the school environment seem to play a slightly more pronounced role compared to the overweight and underweight groups. However, the formal test of network comparison yielded no statically significant differences in structural invariance and global network strength between the normal weight group and adolescents at the lower and upper end of the weight spectrum.

## 4. Discussion

Consistent with the literature e.g., [2,9], the present findings provide clear evidence that overweight adolescents represent a specific risk group for mental health problems. Elevated psychopathological symptoms were observed primarily in the internalizing domain while a similar study in overweight/obese adolescents, which also used the YSR instrument, reported increased psychopathology in both internalizing and externalizing behavioral domains [15]. Most obvious, the eating disorder risk in overweight adolescent was about twice as high as in the normal weight population, which supports previously published literature emphasizing the high prevalence of binge eating and compensatory and unhealthy weight control behavior in overweight and obese adolescent populations [8,49,50]. In this regard, the present results mirror other results in the literature pointing to the shared risk factors for obesity and eating disorders [19,21]. Furthermore, the reduced quality of life scores regarding satisfaction with one’s own body and appearance, relationship to peers and parents and satisfaction with the school environment reported by overweight adolescents and which has been also found in other studies [10,11], points to the urgent need for interventions promoting well-being and mental health in this group.

The question whether underweight adolescents also have an increased risk for mental health problems is much less easy to answer based on the present results. Elevated psychopathological symptoms were found for internalizing problems only (with more socially withdrawn behavior), while quality of life scores were comparable to those of normal weight adolescents and even lower levels of externalizing problems were reported compared to the reference sample. Indeed, whether or not underweight is associated with increased mental health concerns has been controversially discussed in the literature [10,14,51]. As in our study, Drosopoulou et al. [15] reported increased socially withdrawn behavior in underweight adolescents, while they found no differences in other psychopathological symptoms compared to normal weight youth. While underweight is a core characteristic of anorexia nervosa, we found that the eating disorder risk was minimally but significantly lower in underweight compared to normal weight adolescents. This may be surprising; however, a similar result was also found in another large population study where a different instrument to assess eating disorder risk was used [49]. This indicates that underweight per se is not a sign of increased eating disorder risk and that underweight adolescents (provided there are no additional risk factors) would not need specific (preventive) interventions targeting eating disorder symptoms.

The core aim of this study, which also represents the novelty of this research, was the use of psychological network analysis to identify central factors among mental health and well-being variables which inform about potential key targets for interventions for overweight and underweight adolescents. As psychopathological symptoms were most prevalent in overweight adolescents, the following discussion primarily focuses on what can be done for this risk group. The most central variables in the network were anxious/depressed mood and attention problems, while variables associated with eating disorder risk and body dissatisfaction were rather peripheral nodes. At first glance, this seems surprising, but this finding is consistent with another network analysis based on adult individuals with obesity showing that variables directly related to eating disorders were rather placed in the periphery of a network including different psychological characteristics [30]. This may—to some extent—reflect that particularly anxiety problems, but also symptoms of attention-deficit-hyperactivity disorder constitute the most prevalent mental health problems among children and adolescents in general [35,52]. However, it must be emphasized that variables that turn out as most central in psychological network analyses do not necessarily correspond to the most prevalent symptoms of mental health disorders. The main finding from this study (depressive/anxious mood, attention problems as central symptoms in a psychological network, thus representing promising key targets for intervention) contradicts the current practice of many psychological (preventive) interventions among overweight/obese adolescents which primarily aim to reduce disordered eating behavior and weight/shape concerns [53]. Rather, our results indicate that broader intervention approaches, not solely focusing on eating disorder symptoms but (also) incorporating contents to positively impact mood and reduce feelings of depression and anxiety might be most promising. This seems all the more appropriate considering the role emotions and emotion regulation play in individuals with overweight and obesity. Negative affect and stress, for example triggered by weight-related teasing and negative body image, may challenge existing emotion regulation strategies which in turn may result in maladaptive coping such as emotional eating that is often reported in overweight individuals [54,55]. Strengthening skills towards awareness, understanding and acceptance of emotions, self-support and self-compassion may improve resilience, self-efficacy, self-esteem and assertiveness among overweight and obese adolescents [54]. This is in line with a systematic review pointing to the causal link between negative emotions (depression, anxiety, stress) and the development of obesity concluding that adolescents’ anxiety and depression are therefore important targets for preventive interventions of obesity [56].

Apart from anxious/depressed mood, the ‘attention problems‘ subscale of the YSR was also a central variable in the network analysis. On the one hand, this may be linked to the association between attention-deficit-hyperactivity disorder and obesity (e.g., higher levels of impulsivity which may reinforce disregulated eating behaviors) often reported in the literature [57]. Apart from impulsivity and problems with concentration, this scale also assesses inner restlessness and tension. This indicates that intervention components tackling these problems, like the use of relaxation techniques, may be promising concerning promoting well-being in overweight or obese adolescents. This is in line with previous randomized-controlled trials that have shown a beneficial effect of stress management and relaxation intervention (progressive muscle relaxation, guided imagery, diaphragmatic breathing) to reduce general psychopathology, anxiety and depression symptoms in children and adolescents with obesity compared to interventions solely focusing on the change of dietary and physical activity habits [58,59].

Of note, the factors contributing to the development and maintenance of overweight and obesity in childhood and adolescents are manifold as, for example, shown in the ‘Foresight Obesity System Map’ where different biological, medical, psychological, developmental, social and economic factors as well as factors related to diet, physical activity, media and infrastructure have been put together and correlated [60]. The present study provides a contribution to the question of the relative importance of psychological factors in overweight and obesity.

Finally, we found that the network structure of the overweight, underweight and normal weight groups was quite similar. While social problems, socially withdrawn behavior and satisfaction with the school environment tend to play a slightly more central role in the networks of underweight and normal weight compared to overweight adolescents, anxious/depressed mood and attention problems were by far the most important factors within the networks across all groups. Interestingly, in a usability study and survey assessing the adolescents’ and stakeholders’ perspectives on Internet-based prevention for mental health problems in general and for eating disorders and obesity specifically, coping with stress and negative mood were mentioned as the most important topics to address while topics directly related to eating disorders (e.g., healthy nutrition, physical activity) were perceived as less relevant [61,62], which supports the findings of the present study. This has implications for the conceptualization of prevention initiatives in general. Rather than having to focus on different psychological targets for different weight groups, focusing on mood, depression, anxiety and inner restlessness might be promising targets for mental health promotion and preventive interventions across the whole weight spectrum. This is especially important for large-scale interventions to prevent obesity and eating disorders in school settings [63,64], where individualized interventions dependent on the individuals’ weight status are difficult to implement.

The findings from this study must be interpreted in line with the following limitations: First, as in every network analysis, the findings strongly depend on the (variety of) variables that are considered. In this study, we focused on general psychopathological symptoms and well-being variables. Eating disorder symptoms were obtained with a brief screening questionnaire only and we did not obtain detailed information on restraint eating, binge eating or weight/shape concerns using more specific instruments. Due to time constraints, we used the YSR questionnaire assessing different behavioral and emotion problems rather than different instruments assessing different psychopathological symptoms (e.g., depression, anxiety, conduct problems, attention problems) separately (and probably more specifically). Moreover, other (non-psychological) variables like physical activity or dietary habits, which were not addressed in this study, might have provided additional information regarding the interplay between mental health and lifestyle behaviors among overweight and underweight adolescents. Second, weight and height information to calculate the BMI and classify the individual into the overweight, underweight and normal weight groups was obtained via adolescent self-report; thus, these data might lack accuracy to some extent. However, a study based on a general sample of adolescents demonstrated that the difference between self-reported and objectively measured height and weight is marginal [65]. Hence, self-report information should be sufficiently accurate for the purpose of the present study where adolescents were classified into broad weight categories and these data are not used for the diagnosis of anorexia nervosa or obesity. Third, it may be argued that the size of the subsamples of overweight and underweight adolescents may be small given the large number of variables and associations to be estimated. However, to tackle this potential limitation, we used the LASSO estimation which is particularly suitable for smaller samples as it returns a sparse network model where the number of parameters that need to be estimated is reduced [43]. Furthermore, the network stability and edge accuracy measures indicate that the achieved centrality indices and edge weights can be reliably interpreted with the obtained sample size. Finally, it should be noted that we have drawn a community sample of adolescents. Thus, the network analysis primarily informs about promising targets for indicated preventive interventions implemented in community (e.g., school) settings. Future studies may also focus on treatment seeking samples of adolescents with severe obesity or severe underweight which may better inform about important targets for clinical interventions for more severely ill adolescents.

## 5. Conclusions

Psychopathological symptoms and reduced well-being are especially pronounced in overweight or obese adolescents. Thus, stand-alone psychological interventions for this risk group should be considered. At least, mental health components should be an integral part of any intervention for overweight and obese adolescents which primarily focus on promoting healthy dietary and physical activity habits, respectively, concerning losing weight. Our network analysis indicates that psychological interventions focusing on improving mood (respectively, reducing symptoms of depression and anxiety), coping with negative emotions and tackling inner tension, restlessness and stress might be the most promising targets. These variables might also be promising targets for preventive interventions to promote mental health in adolescents across the whole weight spectrum which can be implemented through large-scale school-based initiatives.

## Figures and Tables

**Table 1:** Sample description.

	Overweight Sample (*n* = 344)	Underweight Sample (*n* = 423)	Reference (Normal Weight Sample) (*n* = 1.560)
Female sex (%)	52.6%	66.9%	53.8%
Age (Mean, SD)	14.79 (2.34)	14.30 (2.38)	14.77 (2.27)
Migration background ^1^ (%)	31.7%	22.6%	25.3%
Living with both parents (%)	69.3%	71.3%	74.3%
Residency (living in urban region) ^2^ (%)	54.6%	55.5%	58.8%
Employment status of parents			
Both parents employed (%)	70.2%	77.3%	79.1%
No or one parent employed (%)	29.8%	22.7%	20.9%
BMI (Mean, SD)	27.00 (3.30)	15.67 (1.42)	19.82 (1.64)
BMI-SDS ^3^ (Mean, SD)	1.83 (0.46)	−1.92 (0.63)	−0.01 (0.38)
Any diagnosed psychiatric disorder (%)	8.0%	11.3%	4.0%
Any diagnosed chronic somatic illness (%)	13.3%	14.1%	10.7%
Diagnosed psychiatric disorders in family (%)	6.2%	7.0%	4.0%
Diagnosed chronic somatic illness in family (%)	19.0%	15.4%	16.4%

^1^ Either adolescent or one parent born in a country other than Austria; ^2^ An urban region is defined as living in a city with >10.000 inhabitants; ^3^ SDS = Standard Deviation Score.

**Table 2:** Differences in psychopathology, eating disorder risk and well-being scores of overweight and underweight adolescents compared to a normal weight reference group.

	Overweight Sample (Mean, SD)	Underweight Sample (Mean, SD)	Reference (Normal Weight Sample) (Mean, SD)	Overweight vs. Normal Weight (Tukey Test ^1^)	Underweight vs. Normal Weight (Tukey Test ^1^)
General Psychopathology					
Total Problems	41.06 (23.85)	33.84 (20.72)	34.64 (20.48)	*p* < 0.001	*p* = 0.766
Internalizing Problems	13.16 (11.13)	12.27 (9.21)	11.11 (8.70)	*p* < 0.001	*p* = 0.041
Externalizing Problems	11.22 (6.69)	8.90 (10.47)	10.53 (6.67)	*p* = 0.186	*p* < 0.001
Socially withdrawn	3.46 (2.77)	3.39 (2.73)	2.90 (2.58)	*p* = 0.001	*p* = 0.002
Somatic Complaints	3.50 (3.18)	3.20 (2.82)	3.04 (2.78)	*p* = 0.014	*p* = 0.543
Anxious/Depressed	6.71 (6.48)	6.14 (5.56)	5.56 (5.22)	*p* = 0.001	*p* = 0.115
Social Problems	2.66 (2.44)	2.17 (2.14)	1.99 (2.02)	*p* < 0.001	*p* = 0.264
Thought Problems	1.71 (2.09)	1.41 (1.88)	1.53 (1.93)	*p* = 0.284	*p* = 0.509
Attention Problems	5.10 (3.03)	4.53 (3.07)	4.58 (2.93)	*p* = 0.008	*p* = 0.952
Dissocial Behavior	3.56 (2.53)	2.78 (2.45)	3.36 (2.66)	*p* = 0.412	*p* < 0.001
Aggressive Behavior	7.66 (4.76)	6.12 (4.38)	7.17 (4.66)	*p* = 0.174	*p* < 0.001
Eating Disorder Risk					
SCOFF score	1.33 (1.18)	0.74 (1.07)	0.88 (1.04)	*p* < 0.001	*p* = 0.047
Well-being/Quality of Life					
KIDSCREEN-10	38.08 (7.54)	37.74 (7.19)	40.01 (6.77)	*p* < 0.001	*p* = 0.775
Self-Perception	17.39 (4.60)	19.42 (4.46)	19.10 (4.27)	*p* < 0.001	*p* = 0.321
Parent-Relation & Home-Life	24.90 (5.29)	25.97 (4.59)	25.86 (4.64)	*p* = 0.002	*p* = 0.900
Social support & Peers	16.28 (3.46)	16.68 (17.05)	17.03 (2.92)	*p* < 0.001	*p* = 0.094
School Environment	14.67 (3.57)	15.60 (3.25)	15.31 (3.28)	*p* = 0.004	*p* = 0.283
Social Acceptance/Bullying	13.15 (2.62)	13.99 (1.81)	13.86 (2.01)	*p* < 0.001	*p* = 0.484

^1^ controlled for sex.

## Data Availability

The data that support the findings of this study are available from the corresponding author upon reasonable request.

## Supplementary Materials

The following are available online at https://www.mdpi.com/article/10.3390/nu13114096/s1, Table S1: Standardized centrality indices of the EBIC graphical LASSO network for the overweight and underweight subsamples, Figure S1: Correlation stability plot measuring the stability of betweenness, closeness and strength indices in the overweight subsample, Figure S2: Correlation stability plot measuring the stability of betweenness, closeness and strength indices in the underweight subsample, Figure S3: Edge accuracy plot depicting 95% confidence obtained from 1.000 bootstrap samples drawn from the population overweight adolescents, Figure S4: Edge accuracy plot depicting 95% confidence obtained from 1.000 bootstrap samples drawn from the population underweight adolescents, Figure S5: Network plot and centrality indices plot for adolescents with normal weight.
