# Supplementary material for: Psychopathological Symptoms and Well-Being in Overweight and Underweight Adolescents: A Network Analysis
Source: Nutrients. 2021 Nov 16;13(11):4096. doi: 10.3390/nu13114096 (PMC8624115; doi:10.3390/nu13114096)
Supplement: Supplementary file 1 [file nutrients-13-04096-s001.zip › nutrients-1435138-SI.pdf]

Table S1. Standardized centrality indices of the EBIC graphical LASSO network for the overweight and underweight subsamples.

	**Overweight sample**			**Underweight sample**		
**Variable**	**Betweenness**	**Closeness**	**Strength**	**Betweenness**	**Closeness**	**Strength**
SW	0.181	0.626	0.168	0.726	1.094	0.422
SOM	-0.715	-0.730	-0.383	-0.133	-0.115	-0.163
ANX_DEP	2.721	2.275	2.764	2.157	1.475	2.465
SP	0.032	0.178	0.361	1.012	0.979	-0.013
THOUGHT	-0.715	-0.212	-1.193	-0.991	-0.659	-0.843
ATT	1.526	1.758	1.135	1.298	1.285	1.181
DISS	-0.416	-0.469	0.136	-0.562	-0.463	0.209
AGG	0.480	0.153	0.047	-0.419	-0.233	-0.002
SCOFF	-0.566	-0.786	-0.875	-0.991	-1.031	-0.719
SELF	-0.416	-0.258	-0.582	0.583	0.584	0.219
PARENT	-0.267	-0.242	-0.026	-0.562	-0.198	-0.223
PEERS	-0.864	-1.540	-0.889	-0.991	-1.751	-0.944
SCHOOL	-0.416	-0.595	-0.010	-0.133	0.332	0.155
ACCEPT	-0.566	-0.157	-0.653	-0.991	-1.297	-1.744

Variable Abbreviations: ACCEPT Social Acceptance, AGG Aggressive Behavior, ANX_DEP Anxious Depressed, ATT Attention Problems, DISS Dissocial Behavior, PARENT Parent Relation & Home Life, PEERS Social Support & Peers, SCHOOL School Environment, SCOFF SCOFF Score, SELF Self Perception, SOM Somatic Complaints, SP Social problems, SW Social Withdrawn, THOUGHT Thought Problems.

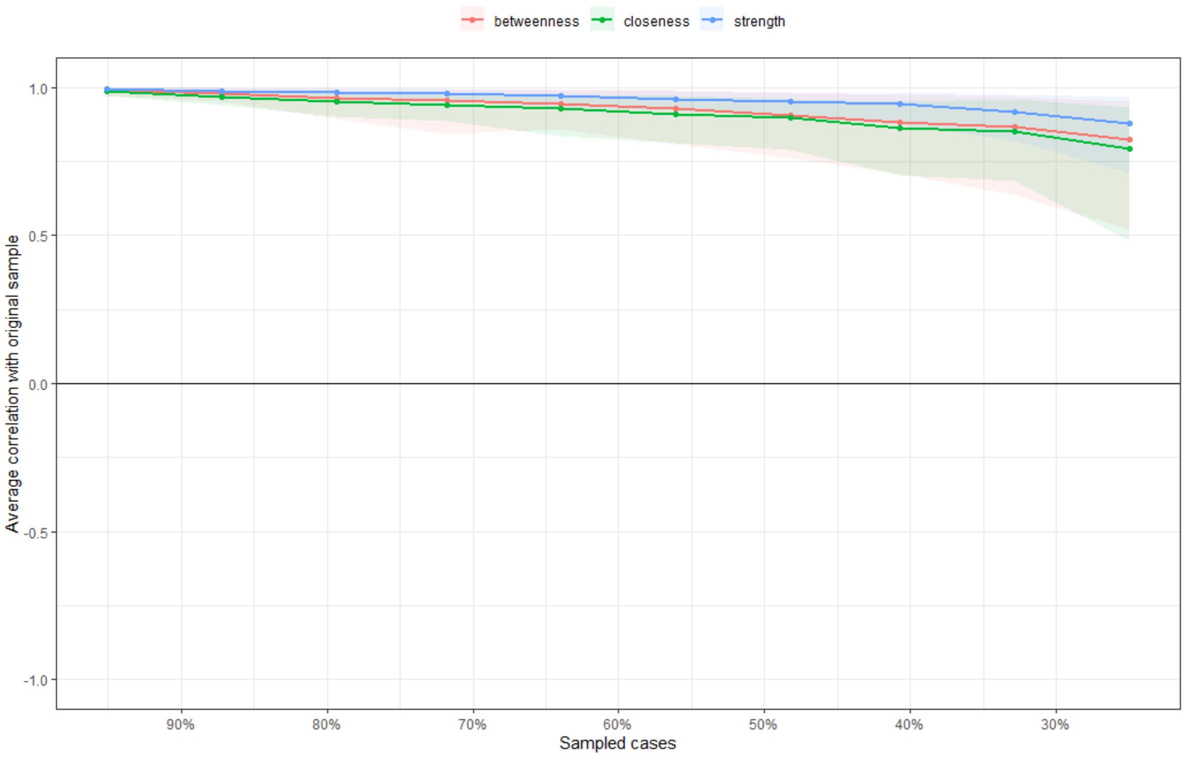

Figure S1. Correlation stability plot measuring the stability of betweenness, closeness and strength indices in the overweight subsample.

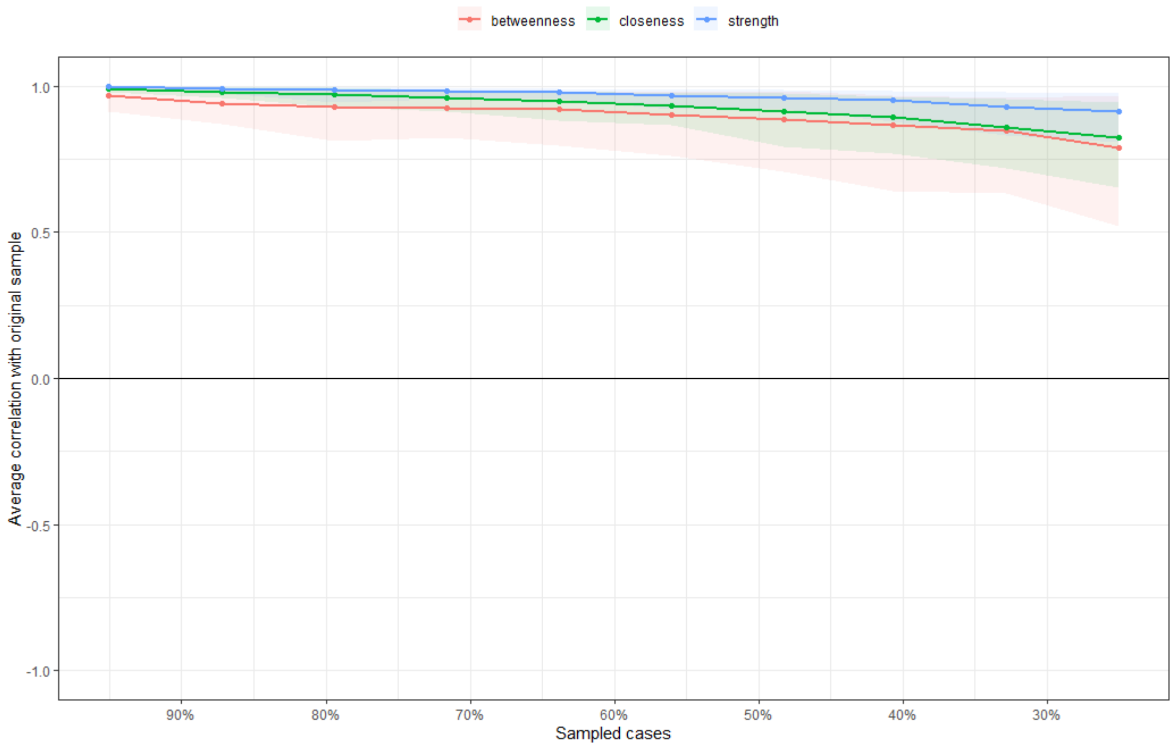

Figure S2. Correlation stability plot measuring the stability of betweenness, closeness and strength indices in the underweight subsample.

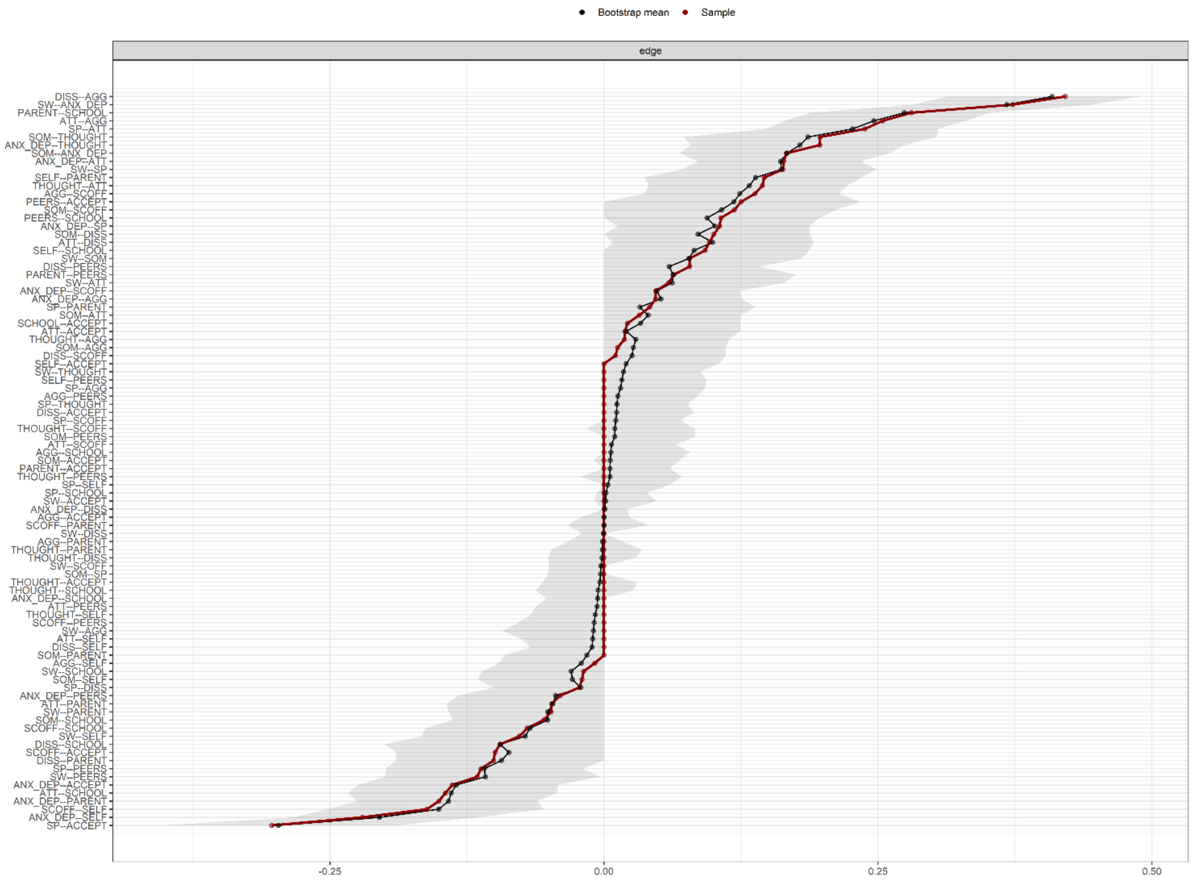

Figure S3. Edge accuracy plot depicting 95% confidence obtained from 1.000 bootstrap samples drawn from the population overweight adolescents.

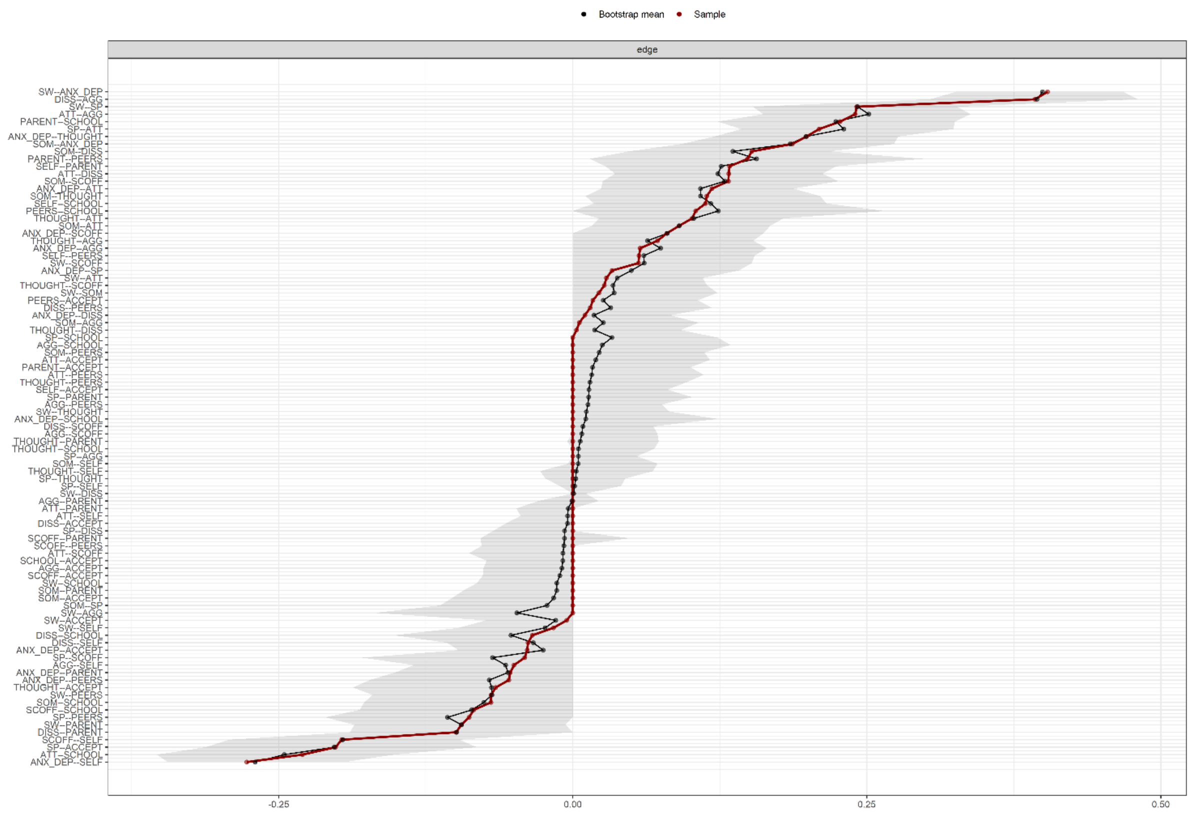

Figure S4. Edge accuracy plot depicting 95% confidence obtained from 1.000 bootstrap samples drawn from the population underweight adolescents.

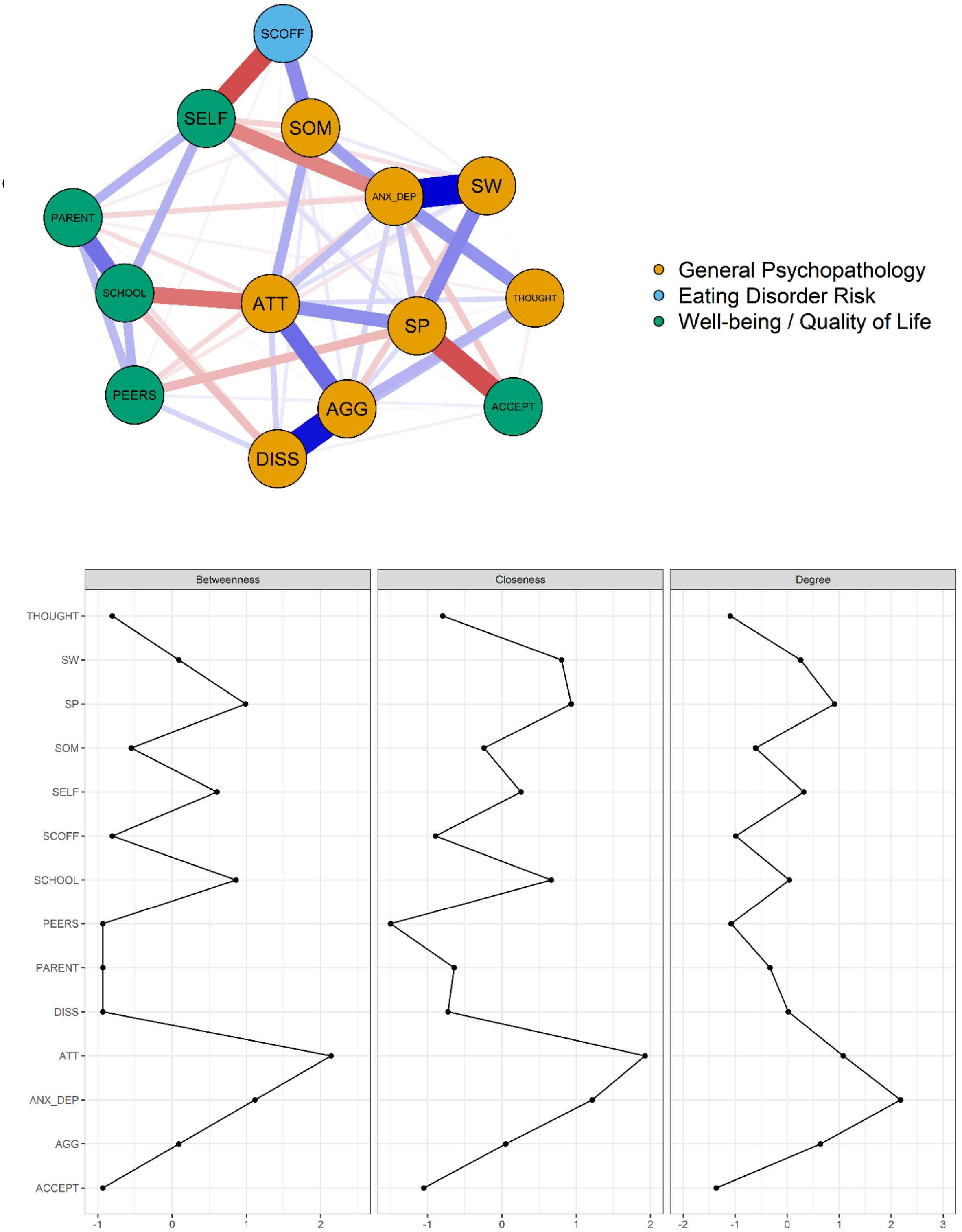

Variable Abbreviations: ACCEPT Social Acceptance, AGG Aggressive Behavior, ANX_DEP Anxious Depressed, ATT Attention Problems, DISS Dissocial Behavior, PARENT Parent Relation & Home Life, PEERS Social Support & Peers, SCHOOL School Environment, SCOFF SCOFF Score, SELF Self Perception, SOM Somatic Complaints, SP Social problems, SW Social Withdrawn, THOUGHT Thought Problems.

Figure S5. Network plot and centrality indices plot for adolescents with normal weight (25th < BMI percentile > 75th).
